# Endoscopic submucosal dissection and JNET classification for colorectal neoplasia: A North American academic center experience

**DOI:** 10.1002/deo2.322

**Published:** 2023-11-27

**Authors:** Nabeel Ahmed, Robert Bechara

**Affiliations:** ^1^ Faculty of Medicine and Health Sciences McGill University Montreal Canada; ^2^ Department of Gastroenterology Kingston Health Sciences Center Kingston Canada

**Keywords:** colorectal neoplasia, endoscopic gastrointestinal surgery, endoscopic submucosal dissection, JNET, narrow‐band imaging

## Abstract

**Objectives:**

Endoscopic submucosal dissection (ESD) enables minimally invasive resection of superficial gastrointestinal neoplasms en bloc regardless of size. The Japan narrow band imaging expert team (JNET) classification utilizes optical magnification and narrow band imaging (NBI) to predict pathology. In North America, ESD is far from ubiquitous, and regional outcomes are not widely described. To date there are no North American studies describing the application and yield of the JNET classification as applied in the practice of ESD.

**Methods:**

A retrospective, single‐center, cohort analysis was performed on a prospectively maintained database of ESD procedures. Between July 2016 and February 2023, all consecutive patients treated with ESD for colorectal lesions were identified and stratified by lesion location, JNET, NBI International Colorectal Endoscopic, lateral spreading tumors, and Paris classifications. Univariate analysis was used for clinicopathological data. *p* < 0.05 was considered statistically significant.

**Results:**

A total of 112 patients were identified. One lesion, a lipoma, was excluded. Overall, 49.5% (55/111) of lesions were colonic and 50.5% (56/111) rectal. Most lesions were lateral spreading tumors (60.4%, 67/111). Overall, 96.4% (107/111) ESDs were successfully completed, 98.1% (105/107) en bloc, and 87.9% (94/107) R0. Adverse events occurred in 1.8% (2/111) of procedures. The median diameter was 4.0 cm and resected in a median time of 62.0 min. Overall, 70.1% (47/67) lesions were upstaged from pre‐ESD biopsy. JNET 2B showed 80.2% (95% CI 71.5–87.1) accuracy for high‐grade dysplasia or sm1. All JNET type 3 were ≥sm2 (*p* < 0.001).

**Conclusions:**

ESD permits safe and effective resection of superficial colorectal neoplasms. JNET classification was more accurate than pre‐resection biopsy at predicting histology in this series.

## INTRODUCTION

Colorectal cancer (CRC) accounts for approximately 10% of cancer diagnoses and related deaths globally, ranking it third in incidence and second in mortality among cancers.^1^ Particularly in Western nations, the incidence of early‐onset CRC is rising and screening and early identification of disease markedly improve survival.^2^ Adenomatous polyps, recognized as precursor lesions to CRC, can be resected endoscopically through various techniques. However, the risk of local recurrence post‐endoscopic resection is a concern, particularly if lesions are resected in a piecemeal fashion.^3^ Endoscopic submucosal dissection (ESD) enables minimally invasive resection of superficial gastrointestinal neoplasms en bloc regardless of size.

**FIGURE 1:**
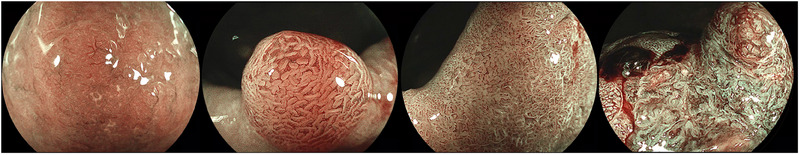
Images of the Japan narrow band imaging expert team (JNET) classification (left to right): JNET 1, JNET 2A, JNET 2B, and JNET 3 lesions.

ESD was first described in Japan in the treatment of gastric cancer in the 1990s and subsequently in colorectal lesions in the early 2000s.^4^, ^5^ Colorectal lesions without deep submucosal invasion and favorable histologic features, such as lack of lymphovascular invasion (LVI), poor differentiation, and tumor budding, can be curatively treated with ESD. Thus, the endoscopic evaluation of lesions to determine suitability for endoscopic resection is paramount. Various classifications, which aim to predict the degree of invasion based on morphologic characteristics, have been proposed. Two such classifications are the narrow band imaging (NBI) International Colorectal Endoscopic (NICE) and Japanese NBI Expert Team (JNET) classifications (Figure 1), which take into account vessel pattern, surface pattern, and color of lesions in order to predict histology.^6^, ^7^, ^8^

NICE classifies lesions into type 1 (hyperplastic/serrated), 2 (adenoma), and 3 (deep submucosal invasive cancer). With lesions that are <1 cm without high‐grade dysplasia (HGD) or sm1 carcinoma, NICE demonstrated the accuracy of 89% in lesions where the prediction was made with diagnostic confidence. Predictions were made with high diagnostic confidence in 75% of cases.^9^ Shortcomings of the NICE classification include the inability to distinguish between low‐grade dysplasia (LGD) and HGD/sm1 lesions as well as the relatively low rate of predictions made with high diagnostic confidence.

In contrast, JNET separates type 2 lesions into types 2A (LGD) and 2B (HGD and sm1 lesions). JNET improves high‐confidence rate to around 92% and offers the ability to differentiate LGD versus HGD to sm1 lesions, predicting the latter with an accuracy of around 80%.^10^ A drawback of JNET is that it requires magnification and it is recommended to use crystal violet for pit‐pattern analysis to improve accuracy for JNET 2B lesions.^11^

In North America, the implementation of ESD in general is far from ubiquitous. Due to the lower prevalence of gastric and esophageal squamous cell carcinoma in North America, the application for ESD has been more focused on colorectal and Barrett's neoplasia, which is more technically challenging.^5^ In Japan, therapeutic endoscopists generally learn ESD first on early gastric cancers and progress to more challenging locations, and so the relative lack in early gastric cancers in North America poses a training hurdle.^12^ Other barriers to the adoption of colorectal ESD in Canada and the United States include longer procedure times and lack of reimbursement for the procedures.^5^, ^13^ Even more sparse in North America is the application of magnifying endoscopy, as evident by the complete lack of publications on the use of JNET.

**FIGURE 2:**
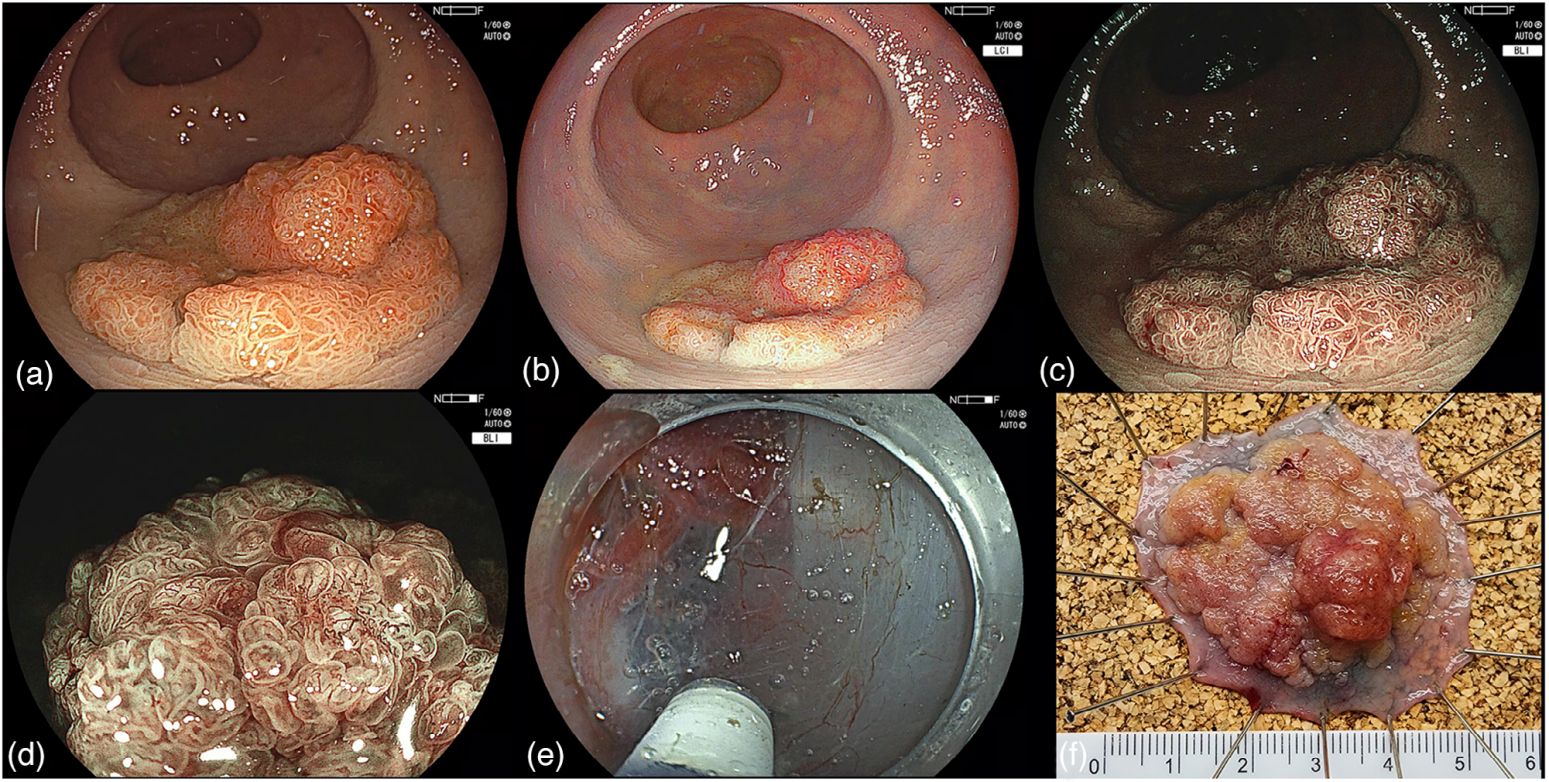
Colorectal endoscopic submucosal dissection of a lateral spreading tumor‐G mixed type with high‐grade dysplasia: (a) white light image, (b) linked color imaging, (c) blue‐light imaging, (d) blue‐light imaging with low magnification Japan narrow band imaging expert team 2B area, (e) Submucosal dissection, and (f) pinned specimen.

Few academic centers in Canada and the United States have published clinical outcomes from their experiences with ESD.^12^, ^14^, ^15^, ^16^ We aim to present outcomes from a single‐center cohort of colorectal ESD conducted at a North American academic center. Additionally, we intend to demonstrate the accuracy of JNET classification, with a particular focus on JNET 2B lesions, which are arguably the optimal lesions to be selected for ESD (Figures 1, 2).

## MATERIALS AND METHODS

### Study design and patient population

This retrospective, single‐center, case series was performed at a single tertiary level referral center in Kingston, Ontario, Canada. Between July 2016 and February 2023, all consecutive patients treated with ESD for colorectal lesions were identified from an administrative database, and charts were retrospectively reviewed. One lesion, a lipoma, was excluded from analysis. Patients who were suspected or proven to have deep submucosal involvement or T2 disease did not undergo ESD. Queen's University Health Sciences & Affiliated Teaching Hospitals Research Ethics Board approval was obtained for the study.

### Variables and data extraction

The primary aim of the study was to describe outcomes from a large single‐center case series of colorectal ESD. The secondary aim was to describe the application of the JNET classification in a North American population. Demographic and clinicopathological variables including ASA score, sex, and initial histologic diagnoses from biopsies, procedure time, and lesion size were extracted from the electronic chart. The procedural variables calculated were efficiency (minutes per cm^2^) and lesion area [area of ellipse = *π* (radius 1)(radius2)]. Depth of submucosal invasion is measured in micrometers and categorized according to the depth of submucosa involved (sm1–3). Superficial (Sm1) was therefore defined as an invasion into the submucosa of 1000 μm or less.^17^ Severe postoperative complications were defined as Clavien Dindo grade ≥2.^18^ Curative resections are defined as R0 resections lacking other high‐risk features such as poor differentiation, histologic LVI, or deep submucosal involvement.^19^

### Procedural technique

All cases were performed by one experienced operator (RB) who completed a formal hands‐on fellowship at Showa University, Tokyo, Japan. The indications used for ESD in our center are as follows: JNET 2B, previously resected lesions that were recurrent, rectal lesions that could not otherwise be removed en bloc, and JNET three lesions if patients refused surgical resection or wanted it done as a diagnostic ESD prior to surgery. Procedures were performed under general anesthesia with endotracheal intubation or with conscious sedation. DualKnife (KD‐650; Olympus), DualKnife J (KD‐655; Olympus), or Flush Knife BTS (DK2620JI; Fujifilm) were used for the procedures. Antithrombotic agents were held in general accordance with American Society for Gastrointestinal Endoscopy recommendations.^20^ The lesions were examined with a combination of white‐light, blue‐light imaging with magnification (up to ×135). JNET and NICE classification were assessed by the same operator at the time of ESD or during diagnostic endoscopy. After submucosal injection was performed, mucosal incision was made, and careful dissection of the submucosa was completed. After complete resection and retrieval, the specimens were pinned down and measured and then fixed in formalin for histopathologic examination (Figure 2). ESD was generally performed on an outpatient basis.

### Statistical analysis

The data was processed using SPSS statistical software (version 27, SPSS Inc.). The data is presented as mean or frequency with range or percentage in parentheses. A univariate analysis was performed using chi‐squared or Fisher's exact test for categorical, whereas continuous variables were compared using the Mann–Whitney *U* test. For all calculations, a *p*‐value of <0.05 was considered statistically significant.

## RESULTS

### Study population

Between July 2016 and February 2023, 112 patients who underwent colorectal ESD were identified. One lesion, a lipoma, was excluded. Baseline demographic and clinicopathological variables of the study population are described in Table 1. Most of the patients were male (67/111, 60.4%) with a median age of 71 years (range 36–93). Most were ASA 3 (66/111, 59.5%). A total of 55 (49.5%) lesions were colonic and 56 (50.5%) rectal. Most lesions were lateral spreading tumors (LSTs) (60.4%, 67/111). LST subtypes were granular homogenous (7.5%, 5/67), granular mixed (56.7%, 38/67), nongranular flat (9.0%, 6/67), and nongranular pseudodepressed (26.9%, 18/67). Most (70.3%, 78/111) were classified as mixed according to the Paris classification. Of those with baseline biopsy pathology, most showed LGD (65.7%, 44/67).

**TABLE 1:** Patient and lesion characteristics.

Variable	*N* = 111 (Median/frequency (range/%))
Gender	
Male	67 (60.4%)
Female	44 (39.6%)
Age (years)	71 (36–93)
ASA	
2	40 (36.0%)
3	66 (59.5%)
4	5 (4.5%)
Non‐LST	44 (39.6%)
LST‐granular homogenous	5 (4.5%)
LST‐granular mixed	38 (34.2%)
LST‐nongranular flat	6 (5.4%)
LST‐nongranular pseudodepressed	18 (16.2%)
Morphology	
Is	16 (14.4%)
Isp	5 (4.5%)
IIa	10 (9.0%)
IIb	1 (0.9%)
IIc	1 (0.9%)
Mixed	78 (70.3%)
Localization	
Colon	55 (49.5%)
Rectum	56 (50.5%)
Prior biopsy pathology (*n* = 67)	
Normal	1 (1.5%)
LGD	44 (65.7%)
HGD	20 (29.9%)
Adenocarcinoma	2 (3.0%)

Abbreviations: HGD, high‐grade dysplasia; LGD, low‐grade dysplasia; LST, lateral spreading tumor.

### Pathologic outcomes

Final pathology is described in Table 2. Lesions were classified histologically as follows: 18.9% (21/111) LGD, 49.5% (55/111) HGD, and 30.6% (34/111) adenocarcinoma. Overall, 16.2% (18/111) showed sm1 invasion, and 15.3% (17/111) showed sm2 invasion or greater. Among lesions with pre‐ESD histology based on biopsy, 70.1% (47/67) lesions were upstaged. Table 6 compares pretreatment biopsy with final pathology results. Among carcinomas, 2.9% (1/34) were poorly differentiated and 17.6% (6/34) showed LVI.

**TABLE 2:** Pathologic outcomes.

Variable	Median/frequency (range/%)
Final pathology	
Low‐grade dysplasia	21 (18.9%)
High‐grade dysplasia	55 (49.5%)
Adenocarcinoma	35 (31.5%)
Depth	
M	76 (68.5%)
Sm1	18 (16.2%)
Sm2	7 (6.3%)
Sm3	6 (5.4%)
T2+	4 (3.6%)
Differentiation	
Well‐moderate	110 (99.1%)
Poor	1 (0.9%)
Histologic features	
Lymphovascular invasion	6 (5.4%)
Tumor budding	5 (4.5%)
Perineural invasion	0 (0.0%)
Upstaged from biopsy (*n* = 67)	47 (70.1%)

### Procedural outcomes

Overall, 96.4% (107/111) ESD were successfully completed, and outcomes are described in Table 3. Overall, 52.3% (58/111) were performed in the endoscopy suite under conscious sedation, with the remaining in the operating theatre under general anesthesia. All procedures were done as outpatient procedures and no patients required admission. All four lesions that were aborted ended up having at least deep submucosal invasion (T2/T2/T2/sm3) were classified as Paris 1s/1s/mixed/mixed, and JNET 2B/2B/3/3. Overall, 98.1% (105/107) were completed en bloc, and 87.9% (94/107) were R0 resections. Overall, 22.4% (24/107) resections were non‐curative (R1 resection, poor differentiation, LVI, or deep submucosal involvement). Overall, 12.1% (13/107) were R1 (12 lateral, 1 deep), one was poorly differentiated, one had LVI+, and 13 were sm2 or greater. Median lesion diameter and area were 4.00 and 10.05 cm^2^, respectively. Lesions were resected with a median efficiency of 6.86 min/cm^2^. Adverse events occurred in 1.8% (2/111) procedures, a delayed bleed, and a microperforation, respectively. The perforation was closed at the time of ESD without clinical sequel, and the delayed bleed was managed conservatively with observation.

**TABLE 3:** Procedural outcomes.

Variable	(Median/frequency (range/%))
Location (*n* = 111)	
Operating theatre	53 (47.7%)
Endoscopy suite	58 (52.3%)
Technical success (*n* = 111)	107 (96.4%)
En bloc resection (*n* = 107)	105 (98.1%)
R0 resection (*n* = 107)	94 (87.9%)
Curative resections (*n* = 107)	83 (77.6%)
Diameter (cm) (*n* = 107)	4.00 (1.1–15.5)
Area (cm^2^) (*n* = 107)	10.05 (0.86–121.74)
Procedure time (*n* = 107)	62.00 (7–230)
Efficiency (min/cm^2^) (*n* = 107)	6.86 (1.60–49.87)
Adverse events (*n* = 111)	
Delayed bleed	1 (0.9%)
Microperforation	1 (0.9%)

### JNET classification

There was a 79.3% concordance with JNET classification. The JNET classification as a function of histology is outlined in Table 4. Most lesions were classified JNET 2B (82.0%, 91/111). Among lesions classified JNET 2B, 12.1% (11/91) showed low‐grade and 58.2% (53/91) demonstrated HGD. Overall, 19.8% (18/91) demonstrated sm1, and 9.9% (9/91) demonstrated ≥sm2 invasion. JNET 2B had a 97.3% (95% CI 90.5–99.7) sensitivity, 47.4% (95% CI 31.0–64.2) specificity, 78.0% (95% CI 72.4–82.8) PPV, 90.0% (95% CI 68.8–97.4) NPV, and 80.2% (95% CI 71.5–87.1) accuracy for HGD or sm1. Submucosal invasion (SMI) was absent in JNET type 1 and 2A and present in 29.7% of type 2B lesions (*p* < 0.001). All JNET type 3 were ≥sm2 (*p* < 0.001).

**TABLE 4:** Japan narrow band imaging expert team classification and pathology.

	JNET 2A	JNET 2B	JNET 3
LGD	10	11	0
HGD	2	53	0
Sm1	0	18	0
Sm2	0	5	2
Sm3	0	2	4
T2+	0	2	2

Abbreviations: HGD, high‐grade dysplasia; LGD, low‐grade dysplasia.

### NICE classification

There was a 79.3% concordance with NICE classification. The NICE classification as a function of histology is outlined in Table 5 . Most lesions were classified NICE 2 (87.4%, 97/111). Among lesions classified NICE 2, 21.6% (21/91) showed low‐grade and 56.7% (55/91) demonstrated HGD. Overall, 16.5% (16/91) demonstrated sm1, and 5.5% (5/91) demonstrated ≥sm2 invasion. All NICE 3 lesions (12.6%, 14/91) had some degree of submucosal invasion. Overall, 14.3% (2/14) were sm1, and the remainder (85.7%, 12/14) were ≥sm2 (*p* < 0.001).

**TABLE 5:** Narrow band imaging International Colorectal Endoscopic (Classification and pathology.

	NICE 2	NICE 3
LGD	21	0
HGD	55	0
Sm1	16	2
Sm2	3	4
Sm3	0	6
T2+	2	2

Abbreviations: HGD, high‐grade dysplasia; LGD, low‐grade dysplasia.

**TABLE 6:** Pretreatment biopsy and pathology.

	Biopsy—LGD	Biopsy—HGD	Biopsy—AdenoCA
LGD	11	1	0
HGD	23	6	0
Sm1	4	8	0
Sm2	3	3	1
Sm3	2	1	1
T2+	1	1	0

Abbreviations: HGD, high‐grade dysplasia; LGD, low‐grade dysplasia.

## DISCUSSION

In this study, we presented our experience in the application of ESD and the JNET classification in the treatment of colorectal lesions in a North American setting. A total of 111 resections were attempted. Four procedures were aborted due to suspicion of deep invasion, all of which were bulky Paris 1s lesions. The final surgical pathology results revealed one sm3, one T3, and two T2 adenocarcinomas. Procedural outcomes were favorable, as 98.1% (105/107) were completed en bloc and 87.9% (94/107) as R0 resections. These rates are similar to those described in Asia from recent meta‐analysis.^3^, ^14^, ^21^ Ultimately, 77.6% (83/107) completed resections were curative, which are similar to rates of curative resection, described in meta‐analysis data.^21^, ^22^

Procedure time in the literature is variable, certainly as a function of lesion size. Our median procedure time of 62.5 min was within the range of what has been described in the literature, whereas our median lesion diameter of 4.00 cm and area of 10.05 cm^2^ were larger than several other study populations.^19^, ^23^, ^24^, ^25^, ^26^, ^27^ Resections were efficient with a median efficiency of 6.86 min/cm^2^, although this metric is not widely reported. Complication rates were low, with a low incidence of perforation (microperforation in 0.9%) and bleeding (delayed bleed in 0.9%). Colonic and rectal subgroups did not differ significantly, except rectal lesions were generally larger (13.2 vs. 6.4 cm^2^, *p* < 0.001) and resected more efficiently (5.0 vs. 7.8 min/cm^2^, *p* = 0.001).

Identifying the group of lesions which are curable by ESD is important. These are generally larger lesions with HGD or superficial submucosal carcinoma without high risk for distant spread. Our results show that pre‐ESD biopsy is not reliable, with over 70% of lesions being upstaged on final pathology compared to the initial biopsy. We specifically evaluated the JNET classification in this context, with a particular focus on JNET 2B, as it is intended to be predictive of HGD or superficial submucosal carcinoma.

We found an overall concordance of 79.3% with the JNET classification. JNET 2B specifically showed 80.2% (95% CI 71.5–87.1) accuracy for HGD or sm1, which parallels what Asian studies have described.^28^, ^29^ Similar proportions of lesions classified as JNET 2B demonstrated either LGD or deep submucosal invasion, but most lesions classified as JNET 2B will be suitable for ESD resection. Furthermore, submucosal invasion was absent in JNET type 2A (*p* < 0.001), and all JNET type 3 had ≥sm2 invasion (*p* < 0.001). Although JNET 2A designation alone does not confirm suitability for ESD, our data suggests that JNET 2B does. This distinction supports the distinction made in the JNET which distinguishes between low‐ and high‐grade/Sm1 lesions as opposed to NICE, which does not. Our data suggests that a JNET 3 designation confirms unsuitability. Excluding JNET 3 lesions would have improved the rate of completed resections (98.1% vs. 96.4%, *p* < 0.001) and the rate of cure among completed resections (81.2% vs. 76.6%, *p* < 0.001).

On the other hand, although all NICE 3 lesions in this series demonstrated some degree of submucosal invasion with a bias toward deep submucosal invasion, 14.3% showed sm1 invasion and would therefore have been amenable to endoscopic resection. Even among NICE 2 lesions, although 78.3% were intramucosal, 5.5% still showed deep submucosal invasion. In this series, the distinctions made in the JNET classification appear to have improved its diagnostic potential as compared to NICE.

As one of the largest reported single‐center North American case series of colorectal ESD, population size is a strength of this study. This study included a fairly comorbid population with relatively large colonic and rectal lesions, which is representative of real‐world North American practice. Despite its merits, there are limitations to our study. Although we had a relatively large number of patients undergoing colorectal ESD, the sample size was not large enough to allow for a multivariable analysis. Additionally, the single‐operator and single‐center nature of this study may limit the generalizability of the findings to a broader North American population.

Opportunities for future research include reporting long‐term outcomes from North American experiences with colorectal ESD and investigating lesion selection criteria for the procedure. As more case series are reported, conducting meta‐analyses that describe regional outcomes and further evaluate the application of the JNET classification will be valuable in advancing our understanding and optimizing the management of colorectal lesions. Development of new techniques for predicting lesion depth, through endoscopic ultrasonography for instance, is ongoing and eagerly awaited.^30^ The development of Western training programs with a focus on optical diagnosis, lesion selection, ESD technique, and addressing logistical barriers to ESD will enable more widespread adoption of the ESD technique in North America.

In conclusion, our North American series has provided evidence confirming the safety and efficacy of ESD for the resection of superficial colorectal neoplasms. Notably, R0 resections were successfully achieved in a majority of patients, and the incidence of complications was low, aligning with outcomes reported in Asian studies. The utilization of the JNET classification proved valuable, demonstrating its efficacy in predicting pathology with superior to pre‐resection biopsy. As colorectal ESD continues to expand in North America, so too should the practice of a detailed endoscopic exam with JNET application for improving lesion selection.

## CONFLICT OF INTEREST STATEMENT

Robert Bechara is a consultant for Olympus, Pentax, Vantage, and Pendopharm.
